# An Exploratory Study of Police Officers: Low Compassion Satisfaction and Compassion Fatigue

**DOI:** 10.3389/fpsyg.2018.02793

**Published:** 2019-01-25

**Authors:** Heath Blair Grant, Cathryn F. Lavery, John Decarlo

**Affiliations:** ^1^Department of Law, Police Science, and Criminal Justice Administration, John Jay College of Criminal Justice, New York, NY, United States; ^2^Department of Criminal Justice, Iona College, New Rochelle, NY, United States; ^3^Department of Criminal Justice, University of New Haven, West Haven, CT, United States

**Keywords:** recovery capital, compassion fatigue, job satisfaction, law enforcement, secondary trauma, substance abuse

## Abstract

**Background:** Compassion fatigue, or the physical, mental, and emotional state experienced by professionals that assist others in distress, has been well documented in several caring professions such as nurses, firefighters, and emergency medical technicians. Until the current study, it has only rarely been examined in police samples despite their high rates of stress and suicide which is a likely result of a depletion of compassion satisfaction, or the pleasure an officer gets from relating to and helping others.

**Aim:** This study documents findings from an ongoing study of compassion fatigue amongst a sample of US urban police officers which suggests the possibility of a future risk for high burnout.

**Conclusion:** Very low levels of compassion fatigue were found in the sampled police officers in comparison to what would be expected from the general population. Where compassion fatigue was found in the sampled police, it was significantly correlated to the level of compassion satisfaction. A potential cause for concern is that the incidence of levels of reported compassion satisfaction were also low in the sample (in the bottom quartile compared to the general population). This suggests a possibility of higher numbers of burnout in the future given the role of compassion satisfaction as a buffer against compassion fatigue in policing.

## Introduction

Compassion fatigue for professionals in care-giving careers has long been recognized. Awareness of this issue for first responders has increased significantly since the 9/11 terror attacks. According to Figley (2002), compassion fatigue is “the cost-of-caring” for those in professions that regularly see and care for others in pain and trauma. Those who work in these fields have either direct exposure to traumatic events (police, emergency hospital workers, nurses, etc.) or even secondary exposure (listening to victims’ experiences, child protection issues, etc.). Compassion fatigue is a potential compelling consequence for anyone who deals persistently with individuals suffering from depression, addiction, poverty or any combination of circumstances that creates hardship or feelings of despair and helplessness (Turgoose et al., 2017). This current exploratory study fills the void in understanding of the incidence of compassion fatigue amongst the much under-studied police profession. It is the first study to test whether or not compassion satisfaction is also negatively associated with burnout in the police profession. This can significantly inform efforts to offer much needed training and other recovery services for police stress and anxiety.

### Compassion Fatigue as a New Perspective in Examining Behaviors of Law Enforcement Officers

When the professional work environment places individuals in consistent states of vulnerability and tension, empathy and compassion can become weakened. This occurs when the practitioner is overstressed, and may result in similar symptoms to those suffered by their clients, victims, etc., (Figley, 2002). Symptoms associated with trauma include: discouragement, difficulty concentrating, hopelessness, cynicism, exhaustion, attrition, and lower levels of job satisfaction. Compassion fatigue is “an occupational hazard” (Mathieu, 2007) because it can lead to toxic work and home environments that also impact those around the practitioner.

Skolnick (1998) describes how a police officer’s “working personality” can impact their perception. It can create an “us versus them” mind-set that is an inevitable byproduct of an inability to “turn-off” the police mentality and experience outside the professional environment. Skolnick focused on certain factors inherent to police training that fuel this working personality, all distinctive to policing: suspicion, dangerousness, authority and social isolation.

Professionals in caregiving fields may not practice enough self-care and can be susceptible to compassion fatigue. Although compassion fatigue has been proposed as a consequence of law enforcement, it is understudied (Cross and Ashley, 2004; Andersen and Papazoglou, 2015). Some of the work done to date has focused on first responders already exhibiting the severe mental health challenges of post-traumatic stress disorder (PTSD) and depression (Andersen et al., 2010).

An individual’s recovery capital is determined by internal and external factors which work in conjunction with one another as an asset in aiding, supporting and sustaining recovery (Berger, 2014). External factors can include: group meetings, family and peer support services or a sponsor, etc. Internal capital reflects the individual’s commitment to recovering from substance abuse, their acknowledgment of their addiction, and attitude toward recovering both what they support internally and project outwardly (Berger, 2014). If an individual’s internal factors are weighted and strong, it helps not only their ability to work through the transitioning external forces but also keeps them grounded and strengthens their resiliency (White and Cloud, 2008; Berger, 2014).

Compassion fatigue is directly related to one’s recovery capital. Spilman’s (2010) trajectory of compassion fatigue for first responders offers a window to how recovery capital might work best with police officers. Much research has demonstrated that new recruits to policing usually enter the profession highly committed with visions of making a difference in the world. Over time, a cynicism has been shown to set in with many that could be the first signs of compassion fatigue. Compassion satisfaction declines with the zombie phase where there is no longer a compassion for the community residents being policed anymore. Together this sets the stage for compassion fatigue in the officer, and its deleterious consequences. If senior law enforcement managers could be trained to recognize these phases, support mechanisms might be established to fortify compassion satisfaction before it turns to burnout.

Stamm (2002) introduced a possible protective factor against the development of compassion fatigue: compassion satisfaction and support. Compassion satisfaction is an internal feeling of increased motivation and satisfaction derived from helping those who suffer. As such, it qualifies as an important form of internal “recovery capital.” Granfield and Cloud (1999) argue that recovery capital includes internal resources that can be used by an individual to get through stress. Recovery capital varies in each person, and directly influences how the person adapts and copes with the challenges they face from substance abuse to anxieties and stress. This affects treatment, sustainability and post-recovery support for the individual. Thus, recovery capital has been linked to a solution-focused therapy to build individual resilience.

Compassion satisfaction is a source of such internal strength that can mitigate the cost of caring spiral that ends with burnout. The social or peer environment of policing is notoriously closed, where the outward display of emotion or empathy can be highly discouraged or seen as a weakness (Miller, 1999). In addition, officers tend to work alone or in a dyad model, unlike firefighters or paramedics who have more of a “team-oriented” mentality. Ultimately, this puts police in a uniquely isolated position, and presents different challenges for senior management, employee assistance professionals and care givers to assist them in distress or crisis. In the cultural environment of police departments, some officers fear repercussions if they share their opinions or if they share feelings of anxiety (Miller, 1999). Under these circumstances, it might not be surprising that compassion fatigue, or feeling less motivated to help alleviate the suffering of others, can surface as a problem.

A new study suggests that there is a significant association between empathy and burnout among officers, but not in the direction one might think (Turgoose et al., 2017). Officers with higher levels of empathy actually had *lower* levels of burnout. Empathy seems to be a form of available recovery capital for officers. Thus, compassion satisfaction could also serve as a buffer against the effects of compassion fatigue because job satisfaction from empathy and caring makes the resulting trauma less severe. In this study the levels of compassion fatigue among the sampled officers overall was not high.

Compassion fatigue can vary by officer demographics as well. Female officers were better at predicting psychological stress and reporting their symptoms. Further analysis conceptualized police stressors as traumatic and routine (Brown et al., 1999).

Burnout is seen as an interrelated component of compassion fatigue along with secondary trauma. Higher levels of compassion fatigue equate to higher levels of secondary trauma, and higher levels of burnout. However, each can be measured as individual components (Stamm, 2010). A 2006 study of child protection workers found that 50% of child protection staff workers suffered from “high” or “very high” levels of compassion fatigue, but the risk of burnout was lower (Conrad and Kellar-Guenther, 2006). Results recommended that compassion fatigue with agency workers needs to be recognized and responded to for successful outcomes with their abused, neglected children and family clients. Adams et al. (2008) research on social workers after the 9/11 terror attacks, clarified the conceptual differences between secondary trauma, burnout and compassion fatigue. Based upon the literature review, we hypothesized that officers would have a significantly higher level of compassion fatigue and lower level of compassion satisfaction than what has been reported in the general population.

## Methods

### Participants and Procedure

The researchers used a convenience sample of 113 responding officers from four participating police departments. Obtaining a sampling frame of officers in each agency from which to randomly select study participants was beyond the scope of the current study but being worked out for a later follow up study. This study did not explore the predictors of compassion fatigue amongst police; the relationships among compassion satisfaction, burnout, and secondary trauma are examined, along with their implications for the field of policing.

Surveys of police officers must be sure to avoid causing the respondents undue stress or harm due to fear of retribution within their agency. Not only would such influences unfairly bias the findings, it would be unethical. As such, the survey was distributed anonymously in select agencies without senior officers present. Subjects were given the surveys in private to be completed without the researchers’ direct participation during administration. This was done to minimize risks and to respect the autonomy of the law enforcement personnel participating (agencies asked that demographic variables not be included for this reason). Only years on the force and rank were collected as variables. The study was approved by the IRB of the University of New Haven (Protocol 2017-001).

### Measures

The Professional Quality of Life Scale (ProQOL; Stamm, 2010) was used in this study because it is the most validated measure in the research literature to measure the effects (positive and negative) of care-giving professions. Importantly, it also separates out compassion satisfaction, which is theorized here to represent potentially practical form of recovery capital for police.

Compassion fatigue is a potential negative aspect of work in helping professions: it is measured through the independent, but interrelated, sub-constructs of burnout and secondary traumatic stress. The shared variance between these scales is 34% (Stamm, 2010). Whereas burnout is associated with negative feelings of hopelessness and feeling like one’s work does not make a difference, secondary traumatic stress involves a fixation on stressful events, and an inability to sleep, and reliving of trauma.

The three dimensions in the ProQOL are independent and do not yield a composite score.

### Data Analysis

SPSS 21.0 was used for the analysis. A comparison of the police officer sample means was made to the general population means reported by Stamm (2010) with the one sample *t-*test.

## Results

### Prevalence of Compassion Satisfaction, Secondary Traumatic Stress, and Burnout

Sampled police officers did not demonstrate compassion fatigue. The average scores on both burnout and secondary trauma were significantly below (*p* < 0.000) the general population (see Table 1). This is seemingly a positive finding indicating low fatigue and burnout amongst the sampled officers.

**Table 1:** Comparison of police officers with the general population (one sample *t*-test).

	*t*	*df*	Sig (2 tailed)
Burnout	–56.667	111	0.000
Secondary Trauma	–56.886	108	0.000
Compassion Satisfaction	–20.864	112	0.000


*Test value = 50.*

### Bivariate Relationship Between Compassion Satisfaction, Burnout, and Secondary Trauma Amongst Police Officers

Table 2 provides the bivariate correlations between the three ProQOL scales. Consistent with the research literature, compassion satisfaction is significantly (*p* < 0.01) negatively associated with burnout (*p* < 0.000) and secondary traumatic stress (*p* < 0.000) in the current study’s sample of police, reflecting the potential of compassion satisfaction to be both a protective factor and form of recovery capital for police officers.

**Table 2:** Burnout, secondary trauma, and compassion satisfaction for police officers: correlations between the variables.

	Burnout	Secondary Trauma	Compassion Satisfaction
Burnout	1	0.739^∗^	–0.597^∗∗^
Secondary Trauma	0.739^∗^	1	–0.235^∗^
Compassion Satisfaction	–0.597^∗∗^	–0.235^∗^	1


*^∗^Correlation is significant at the 0.05 level (2 tailed). ^∗∗^Correlation is significant at the 0.01 level (2 tailed).*

## Discussion

The current study fills a gap in the research literature by applying the growing research on compassion fatigue and recovery capital in other helping professions to the law enforcement profession. On a descriptive level the current study replicates Turgoose et al. (2017) finding that levels of compassion fatigue and burnout amongst their sample of police officers was not high. Turgoose et al. (2017) also found that burnout was not associated with high levels of empathy. To the contrary, higher levels of empathy were associated with less burnout. Amongst police officers, compassion satisfaction was very significantly associated with less burnout and stress, supporting its possible role as recovery capital for police.

Gaining job satisfaction from the helping aspects of policing, such as empathy, can be a form of recovery capital. Further research must be completed to examine the causal pathway more closely. Current research made no attempt to look at causal predictors of either compassion satisfaction or compassion fatigue as with other helping professions. Although no significant differences in these variables have been reported by race and other demographics in other helping professions (Stamm, 2010) this needs study within the police profession as well.

The fact that the two studies done in this area have not found a high incidence of compassion fatigue might be a positive finding given that it is so contrary to expectations. Future research should see findings hold up in higher crime areas, or communities with significant police/community tensions or low legitimacy. Compassion satisfaction could be negatively impacted by having to help community residents that are belligerent, or outwardly antagonistic toward helpers. Although police agencies in the current study were all urban, many were in low crime areas of New York State and Connecticut. This may be driving a low incidence of compassion fatigue.

### The Fact That the Officers Are in the Bottom Quartile for Compassion Satisfaction Should Receive Significant Attention

With this sample, compassion satisfaction is very negatively associated with burnout and secondary trauma (compassion fatigue). Although there are low levels of burnout and secondary trauma found in this cross-sectional study, it may be a canary in the mine for future burnout and trauma if not addressed. Although deserving of future study to iron out the true causal pathway and predictors of compassion fatigue, there is enough established to warrant caution and research attention.

These findings have significant implications. Officers are often encouraged to restrict their emotions both in the field and with other officers. Agencies might consider providing more opportunities for officers to share experiences in the field to guard against this and its possible link to later compassion fatigue. According to Miller et al. (2009), opportunities for such feedback are a significant predictor of job satisfaction. Officers reported higher levels of job satisfaction when feedback was available from supervisors and colleagues on their overall performance. Compassion satisfaction can be pivotal in helping law enforcement appreciate the value of their services within their communities.

## Author Contributions

CL assisted in data collection and the writing of the article. HG is the first author and study founder. JD contributed to IRB approval and data collection.

## Conflict of Interest Statement

The authors declare that the research was conducted in the absence of any commercial or financial relationships that could be construed as a potential conflict of interest.
